# ATP-Dependent Chromatin Remodeling by Cockayne Syndrome Protein B and NAP1-Like Histone Chaperones Is Required for Efficient Transcription-Coupled DNA Repair

**DOI:** 10.1371/journal.pgen.1003407

**Published:** 2013-04-18

**Authors:** Iltaeg Cho, Pei-Fang Tsai, Robert J. Lake, Asjad Basheer, Hua-Ying Fan

**Affiliations:** Epigenetics Program, Department of Biochemistry and Biophysics, Department of Genetics, Perelman School of Medicine, University of Pennsylvania, Philadelphia, Pennsylvania, United States of America; University of Washington, United States of America

## Abstract

The Cockayne syndrome complementation group B (CSB) protein is essential for transcription-coupled DNA repair, and mutations in CSB are associated with Cockayne syndrome—a devastating disease with complex clinical features, including the appearance of premature aging, sun sensitivity, and numerous neurological and developmental defects. CSB belongs to the SWI2/SNF2 ATP–dependent chromatin remodeler family, but the extent to which CSB remodels chromatin and whether this activity is utilized in DNA repair is unknown. Here, we show that CSB repositions nucleosomes in an ATP–dependent manner *in vitro* and that this activity is greatly enhanced by the NAP1-like histone chaperones, which we identify as new CSB–binding partners. By mapping functional domains and analyzing CSB derivatives, we demonstrate that chromatin remodeling by the combined activities of CSB and the NAP1-like chaperones is required for efficient transcription-coupled DNA repair. Moreover, we show that chromatin remodeling and repair protein recruitment mediated by CSB are separable activities. The collaboration that we observed between CSB and the NAP1-like histone chaperones adds a new dimension to our understanding of the ways in which ATP–dependent chromatin remodelers and histone chaperones can regulate chromatin structure. Taken together, the results of this study offer new insights into the functions of chromatin remodeling by CSB in transcription-coupled DNA repair as well as the underlying mechanisms of Cockayne syndrome.

## Author Summary

Cockayne syndrome is a devastating inherited disease; the average life span of those afflicted is 12 years. Cockayne syndrome patients have features of premature aging, are highly sensitive to sunlight, and suffer from numerous developmental and neurological disorders. The majority of Cockayne syndrome patients have mutations in the CSB protein; however, how these mutations can lead to Cockayne syndrome is largely unknown. CSB is essential for transcription-coupled DNA repair—a process that preferentially removes bulky DNA lesions that stall transcription, such as those created by ultraviolet light. In eukaryotes, DNA is packaged into nucleosomes, which consists of DNA wrapped around a set of core histone proteins, and nucleosomes can create barriers to the DNA repair process. In this study, we found that CSB can slide histones along DNA. We also found that histone chaperones, proteins that accept and donate histones, greatly facilitate this process. Importantly, we show that CSB derivatives that are unable to move nucleosomes or collaborate with histone chaperones cannot repair UV-induced DNA lesions. Our study reveals that nucleosome remodeling by CSB is important for transcription-coupled DNA repair and suggests that an inability to efficiently mobilize nucleosomes might contribute to the underlying mechanism of Cockayne syndrome.

## Introduction

Cockayne syndrome is an autosomal recessive disease associated with numerous developmental and neurological defects, sun sensitivity and the appearance of premature aging. More than 70% of Cockayne syndrome patients have mutations in the locus encoding the Cockayne syndrome complementation group B (CSB) protein. CSB is important for transcription regulation mediated by RNA polymerase I, II and III, and it is crucial for transcription-coupled DNA repair [1]–[4]. CSB is also implicated in base-excision DNA repair [5], [6].

The CSB protein belongs to the SNF2/SWI2 ATP-dependent chromatin remodeling protein family. Members of this family regulate chromatin structure non-covalently, by using ATP as energy to alter histone-DNA contacts. Depending upon the particular chromatin remodeler, these alterations can lead to changes in nucleosome position, composition or conformation [7], [8]. However, not all members of this broad protein family have been shown to possess chromatin-remodeling activity. A collection of biochemical activities has been demonstrated for the CSB protein, including DNA/nucleosome-stimulated ATP hydrolysis, DNA strand annealing and exchange, as well as DNA wrapping [9]–[12]. Furthermore, CSB was shown to alter DNase I hypersensitivity of mononucleosomal DNA and randomize a nucleosomal array [13]. However, the extent to which CSB alters nucleosome structure, the nature of the remodeled products and the biological significance of ATP-dependent chromatin by CSB remain largely undescribed. Currently, *in vivo*, it is only known that the DNA/nucleosome-stimulated ATPase activity of CSB is essential for the recruitment of CSB to lesion-stalled transcription, a critical and early step of transcription-coupled DNA repair [14].

ATP-dependent chromatin remodelers often complex with other proteins [8]. These non-catalytic subunits are involved in targeting the respective remodeling activity and in regulating the activity of the motor protein. Although CSB has been found to interact with several proteins, how these interactions impact CSB's activities is still unclear. Interestingly, CSB exhibits auto-regulation, in which the N-terminal region negatively regulates both the ATPase activity and stable CSB-chromatin association [14].

Histone chaperones are also important for chromatin structure regulation [15]. These proteins function as histone carriers, donating and accepting histones. *In vitro*, histone chaperones can coordinate their activities with ATP-dependent chromatin remodelers to facilitate chromatin assembly or disassembly. For example, ACF or CHD1 can work with the NAP1 chaperone in chromatin assembly [15], [16], while yeast RSC can cooperate with NAP1 in nucleosome disassembly [17].

The human genome encodes five NAP1-like proteins (NAP1L1–NAP1L5) that share homology with yeast NAP1, the first NAP1 cloned [18]. NAP1L1 and NAP1L4 are ubiquitously expressed, while NAP1L2, NAP1L3 and NAP1L5 are predominantly expressed in neurons [19]. These latter three proteins are believed to have evolved more recently and likely perform mammalian-specific functions (Figure S1A) [20]. NAP1L1 and NAP1L4 are more ancient and share ∼63% amino acid identity (Figure S1B) [21]. The most notable difference between NAP1L1 and NAP1L4 is that the former has three clusters of acidic residues [22] while the latter has only two [21]. Both NAP1L1 and NAP1L4 possess histone chaperone activity and are believed to have similar functions [23]. Structure studies predict that the NAP1-like proteins can heterodimerize. Other members of the NAP1 family include SET, CINAP and TSPY [19].

In this study, we used a collection of *in vitro* and *in vivo* assays to characterize CSB's remodeling activity and to dissect the functions of CSB in transcription-coupled DNA repair. We demonstrate that CSB can reposition nucleosomes and that this activity is greatly enhanced by NAP1-like histone chaperones. Moreover, we show that chromatin remodeling, orchestrated by the combined activities of CSB and the NAP1-like chaperones, is required for efficient transcription-coupled DNA repair. From our results, we present a model in which CSB has two separable functions in transcription-coupled DNA repair: one function is protein factor recruitment and that is independent of nucleosome remodeling. A second activity, which is dependent upon nucleosome remodeling, is to establish a chromatin environment that is conducive for DNA repair and/or transcription resumption after repair.

## Results

### CSB Remodels Nucleosomes in an ATP–Dependent Manner

**Figure 1:**
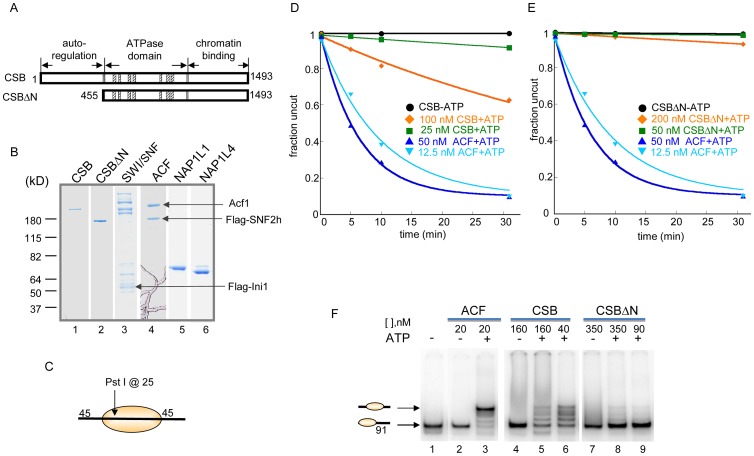
ATP–Dependent Chromatin Remodeling Activity of CSB. (A) Schematic representation of CSB and CSBΔN proteins used in the remodeling assays shown in D–E. (B) Coomassie-stained gel showing purified proteins and complexes used in this study. (C) Representation of the centrally localized mononucleosome with two 45 bp DNA overhangs used in assays shown in D–E. The position of the Pst I site used to assay restriction enzyme accessibility is as noted. (D–E) Restriction enzyme accessibility assays to examine the kinetics of remodeling. Rate constants for ACF at 12.5 and 50 nM were 0.1 and 0.16 min^−1^, respectively. (D) Rate constants for CSB at 25 and 100 nM were 2.5 and 17×10^−3^ min^−1^, respectively. Rate constant for CSB in the absence of ATP was 4×10^−7^ min^−1^. (E) Rate constants for CSBΔN at 50 and 200 nM were 3 and 22×10^−4^ min^−1^, respectively. In the absence of ATP, the rate constant for CSBΔN was 2.5×10^−4^ min^−1^. (F) Electrophoretic mobility-shift assays to analyze remodeled nucleosome structure. End-positioned mononucleosomes with a single 91-bp DNA overhang were used as substrate. Reactions were carried out for 15 minutes at 30°C, as previously described [26]. ACF created a centrally localized nucleosome, as previously reported [25]. CSB and CSBΔN were used at two concentrations, as indicated. Migration positions of centrally- and end-positioned nucleosomes are as noted.

To determine the extent to which CSB functions as an ATP-dependent chromatin remodeler, we determined the rate at which a restriction site that is occluded by a histone octamer could be made accessible by CSB. To accomplish this, we performed quantitative, side-by-side restriction enzyme accessibility assays comparing the rate of site exposure by CSB to that of ACF, a well characterized, robust ATP-dependent chromatin remodeling complex (Figure 1A–1B). We used as substrate in these assays a nucleosome composed of a 240 bp DNA fragment, with a histone octamer positioned at its center and a single Pst I site located within the nucleosomal DNA, 25 bp away from the entry/exit site (Figure 1C) [24]. While ACF made nucleosomal DNA accessible for Pst I cutting at a maximal rate of ∼0.13 min^−1^, 100 nM CSB remodeled nucleosomes at a rate approximately 10-fold slower (∼0.017 min^−1^) (Figure 1D). Approximately 60% of the Pst I site was still inaccessible for Pst I cutting after remodeling by 100 nM CSB for 30 min; in contrast, less than 10% of the Pst I site was inaccessible after remodeling by ACF for the same length of time.

The requirement for free, linker DNA that flanks a core nucleosome varies between ATP-dependent chromatin remodelers and, consequently, linker DNA length can impact remodeling activity. We, therefore, assayed CSB remodeling of mononucleosomal substrates with different linker lengths. As shown in Figure S2, a linker of ∼120 bp, on either or both sides of a core nucleosome, did not significantly enhance nucleosome remodeling by CSB. In addition, among all mononucleosomal substrates tested, CSB remodeled core mononucleosome the slowest. Taken together, these quantitative restriction enzyme accessibility assays revealed that CSB is a relatively inefficient remodeling enzyme on its own.

### The N-Terminal Region of CSB Couples ATP Hydrolysis to Chromatin Remodeling

Previously, we found that the N-terminal region of CSB prevents the stable association of CSB with chromatin in the absence of UV damage and, therefore, functions in auto-regulation. We next determined how the N-terminal region of CSB contributes to the process of chromatin remodeling by examining the remodeling activity of CSBΔN (Figure 1).

As shown in Figure 1E, deleting the N-terminal 454 amino acids substantially diminished the ability of CSB to make nucleosomal DNA accessible for Pst I cutting. The decreased remodeling activity was not due to an inability to hydrolyze ATP, as CSBΔN was still a functional DNA- and nucleosome-stimulated ATPase (Figure S3) [10], [14]. These results revealed that the N-terminal 454 amino acids of CSB are important to couple ATP hydrolysis to chromatin remodeling.

### CSB Repositions Nucleosomes in an ATP–Dependent Manner

The restriction enzyme accessibility assays described above demonstrated that CSB could expose a DNA site occluded by a histone octamer. We next used native polyacrylamide gel electrophoresis to examine the structure of CSB remodeled products (Figure 1F). Using a mononucleosome with a 91-bp DNA overhang as substrate (lane 1), we found that CSB created an array of remodeled products with different mobilities, representing nucleosomes of different translational positions (lanes 5–6). As shown previously and in Figure 1F, ACF also altered the translational position of a histone octamer in an ATP-dependent manner, but this remodeling complex created a single, predominant product composed of a centrally localized nucleosome (lane 3) [25].

Consistent with results obtained from restriction enzyme accessibility assays, in the absence of the N-terminal region, CSB only slightly changed the mobility of the mononucleosomal substrate (Figure 1F, lanes 8–9). Therefore, CSBΔN possesses little remodeling activity (Figure 1E), supporting the notion that the N-terminal region of CSB couples ATP hydrolysis to chromatin remodeling.

### Histone Chaperones NAP1L1 and NAP1L4 Are New CSB Binding Partners

**Figure 2:**
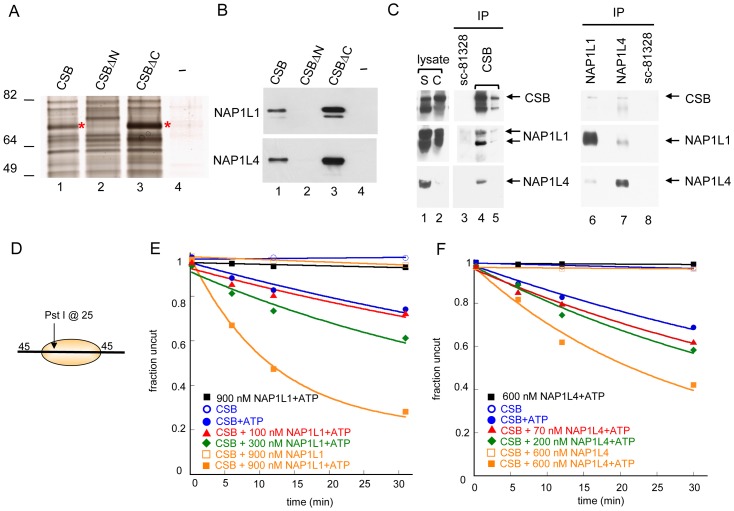
Histone Chaperones NAP1L1 and NAP1L4 Interact with CSB and Enhance ATP–Dependent Chromatin Remodeling by CSB. (A) Silver stained gel resolving proteins from UV-irradiated CS1AN-Sv cell lysates that bound to immobilized CSB, CSBΔN (CSB 455–1493), CSBΔC (CSB1-1009) or an M2-beads-only control (−). Asterisks denote bands excised for analysis by mass spectrometry. (B) Western blot analyses of samples shown in (A), using anti-NAP1L1 or NAP1L4 antibodies. (C) Co-immunoprecipitation of CSB, NAP1L1 and NAP1L4 from chromatin-enriched fractions of UV-irradiated HeLa cells (S is the soluble fraction, and C is the chromatin-enriched fraction). Antibodies were crosslinked to beads and co-immunoprecipitating proteins were eluted. The first two elutions are shown (lane 4–5). Western blots were performed with antibodies indicated to the right. (D) Mononucleosome used in assays E–F. (E–F) Restriction enzyme accessibility assays with 280 nM CSB. (E) Effect of NAP1L1 on CSB remodeling activity. Rate constants for CSB with 0, 100, 300 and 900 nM NAP1L1 were 1, 1, 2 and 6, ×10^−2^ min^−1^, respectively. In the absence of ATP or CSB, no appreciable stimulation of activity was detected. (F) Effect of NAP1L4 on CSB remodeling. Rate constants for CSB with 0, 70, 200 and 600 nM NAP1L4 were 1, 2, 2, and 4, ×10^−2^ min^−1^, respectively. In the absence of ATP or CSB, no appreciable stimulation of activity was detected.

CSB remodeled nucleosomes in an ATP-dependent manner, but with 10-fold less efficiency when compared to ACF (Figure 1D). As ATP-dependent chromatin remodelers often cooperate with other proteins in chromatin remodeling, we sought to identify proteins that interact with CSB and enhance its remodeling activity (Figure 2). To accomplish this, whole cell lysates were prepared from UV-irradiated CSB-deficient cells (CS1AN-Sv) and incubated with immobilized Flag-CSB, Flag-CSBΔN or Flag-CSBΔC (CSB1-1009), purified from SF9 cells [14]. Proteins that bound to CSB, CSBΔN or CSBΔC were eluted, resolved by SDS-PAGE and silver stained. Strikingly, a notable difference in protein binding was observed (Figure 2A, asterisks); this CSB-interacting protein bound to both CSB and CSBΔC, but not to CSBΔN. Mass spectrometry revealed that these protein bands contained the human NAP1-like histone chaperones, NAP1L1 and NAP1L4 (Figure S4A–S4B). To ensure that the peptides recovered by mass spectrometry were truly unique to NAP1L1 and NAP1L4, we compared them to the non-redundant protein sequences deposited in the NCBI database. We found that these peptides only matched to human NAP1L1 and NAP1L4, but not other NAP1-like proteins. Western blot analysis using the material isolated from these binding assays confirmed that NAP1L1 and NAP1L4 bound to CSB and that the N-terminal 454 amino acids of CSB was crucial for this interaction (Figure 2B).

To determine if CSB interacts with NAP1L1 and NAP1L4 *in vivo*, we immunoprecipitated endogenous CSB, NAP1L1 and NAP1L4 individually from chromatin-enriched protein fractions of UV-treated HeLa cells, and used western blot analysis to identify co-purifying proteins (Figure 2C). As a negative control, we used an anti-NAP1L1 antibody (SC-81438) that did not immunoprecipitate NAP1L1 under our experimental conditions. As shown in Figure 2C, both NAP1L1 and NAP1L4 co-purified with CSB after UV irradiation. Reciprocally, western blot analysis of anti-NAP1L1 or NAP1L4 immunoprecipitates revealed that CSB co-purified with both these proteins. Moreover, the interaction between CSB and NAP1L1 occurred independently of UV irradiation, although this interaction was slightly enhanced after cells were exposed to UV (Figure S4C). These results revealed that CSB interacts with the histone chaperones NAP1L1 and NAP1L4 both *in vitro* and *in vivo*.

### NAP1-Like Histone Chaperones Enhance Chromatin Remodeling by CSB

To elucidate the functional significance of the interaction between CSB and NAP1-like histone chaperones, we determined the effect of NAP1L1 and NAP1L4 on CSB-mediated chromatin remodeling. Using restriction enzyme accessibility assays, we found that NAP1L1 or NAP1L4 increased the kinetics of CSB remodeling in a dose-dependent manner, reaching a remodeling rate close to the maximal rate of ACF (Figure 1D and Figure 2E–2F). Importantly, under our experimental conditions, NAP1L1 and NAP1L4 did not remodel nucleosomes on their own, even at very high concentrations (900 nM and 600 nM, respectively) (Figure 2E–2F). In all cases, nucleosome remodeling was dependent upon the presence of CSB and ATP. Together, these results revealed that CSB cooperates with the histone chaperone NAP1L1 or NAP1L4 to achieve robust ATP-dependent chromatin remodeling activity.

### CSB and NAP1-Like Chaperones Create a Centrally Localized Nucleosome as the Predominant Remodeled Product

**Figure 3:**
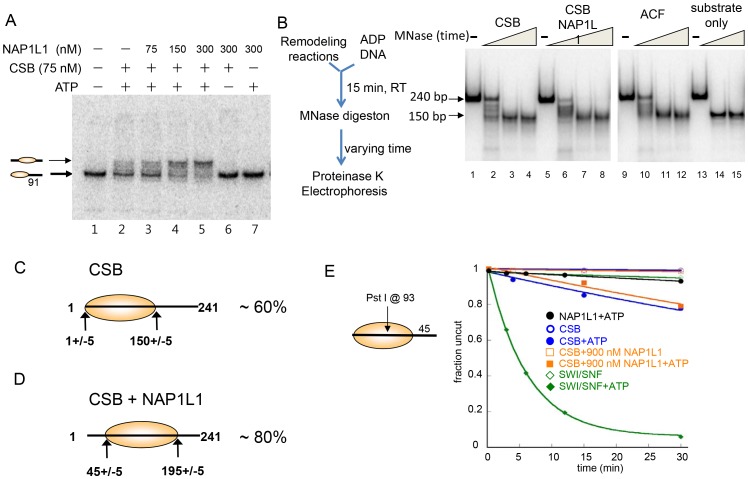
CSB and NAP1-like Chaperones Efficiently Slide Nucleosomes to the Center of a DNA Fragment. (A) Electrophoretic mobility-shift assays to analyze remodeled nucleosome structure. An end-positioned mononucleosome with a 91 bp DNA overhang was used as substrate. Varying amounts of NAP1L1 were used in CSB-mediated nucleosome remodeling assays. NAP1L1 facilitates chromatin remodeling by CSB to form centralized nucleosomes. Nucleosome positions are illustrated to the left. (B) MNase mapping of protected DNA before and after remodeling reactions. Schematic representation of the assay is to the left. (C) Results from MNase mapping. After CSB remodeling, ∼60% of the nucleosomes were similar to the end-positioned nucleosomal substrate. (D) After remodeling by CSB and NAP1L1, ∼80% of the nucleosomes were centrally positioned. (E) Restriction enzyme accessibility assays using a 202 bp nucleosomal substrate with a Pst I site at position 93.

We next used native polyacrylamide gel electrophoresis to examine the structure of the remodeled products generated by the concerted action of CSB and NAP1L1 (Figure 3A). In the absence of NAP1L1, CSB created an array of remodeled products. By adding NAP1L1 into the reactions, one predominant remodeled product was generated with a mobility similar to that of a centrally localized nucleosome, such as that generated by ACF (Figure S5A). Similar results were obtained with NAP1L4 (data not shown). Consistently, CSB and the NAP1-like proteins did not move a centrally localized nucleosome.

To determine if the remodeled products generated by CSB alone or by CSB and NAP1L1 were canonical nucleosomes composed of ∼150 bp of DNA wrapped around a histone octamer, we used limited micrococcal nuclease (MNase) digestion to determine the length of protected DNA in the remodeled products. As shown in Figure 3B, the remodeled products generated by CSB or by the combined activities of CSB and NAP1L1 contained an MNase-resistant DNA fragment of ∼150 bp in length, indicating that CSB creates canonical nucleosomes.

Restriction enzyme mapping of the 150 bp MNase-resistant DNA fragments isolated by gel purification (Figure 3B, lane 4) revealed that about 60% of the nucleosomes in the CSB remodeled products contained end-positioned nucleosomes, identical to the substrate; the remaining nucleosomes contained histone octamers at different translational positions (Figure 3C and Figure S5B-S5C). On the other hand, mapping the remodeled products generated by CSB and NAP1L1 (Figure 3B, lane 8) revealed that more than 80% of the remodeled product contained a centrally localized nucleosome (Figure 3D and Figure S5B-S5C), as was predicted from the mobility of the remodeled nucleosomes in a native gel (Figure 3A). Together, these results demonstrated that the remodeled products generated by CSB or by CSB and NAP1L1 are canonical nucleosomes. Furthermore, these results suggest that CSB displays nucleosome-centralizing activity and that the NAP1-like proteins make this activity more apparent.

ATP-dependent chromatin remodelers such as SWI/SNF not only alter nucleosome positions but can also alter nucleosome composition and conformation [26], [27]. To dissect further the mechanisms by which CSB and NAP1L1 remodeled nucleosomes, we determined whether NAP1L1 could help CSB render DNA sites located near the nucleosome dyad (the nucleosome center) accessible for restriction enzyme cutting. If the nucleosome contains a 45-bp DNA linker, then nucleosome sliding will not efficiently expose a site near the dyad, as this site would always lie within the bounds of the histone octamer. Using as substrate a 202 bp nucleosome with a single Pst I site located at position 93, we found that SWI/SNF efficiently exposed position 93 at a rate of 0.16 min−1 (Figure 3E), consistent with the previous finding that SWI/SNF-remodeled products can have less than 147 bp of DNA protected by the histone octamer [27]. CSB exposed this site very inefficiently, at a rate 20-fold less than SWI/SNF (0.008 min^−1^). Moreover, NAP1L1 did not enhance the ability of CSB to expose this site (Figure 3E). These results demonstrated that, together, CSB and NAP1L1 efficiently expose DNA near the entry/exit sites of a nucleosome (Figure 2E), but not near the dyad (Figure 3E). These observations are also consistent with the propensity of CSB to generate centered nucleosomes, and CSB does not appear to be able to slide nucleosomes off DNA ends.

### A Conserved Basic Domain in the N-Terminal Region of CSB Mediates NAP1L1 Interaction and Is Required for Robust ATP–Dependent Chromatin Remodeling Activity

To begin to elucidate the contribution of CSB's chromatin remodeling activity to the process of transcription-coupled DNA repair, we sought to generate a CSB derivative that would allow CSB to be recruited to chromatin in a DNA-lesion-dependent manner but, at the same time, be defective for efficient chromatin remodeling. We focused our attention on the N-terminal region of CSB, as this region is important for chromatin remodeling (Figure 1, Figure 2) and UV-induced CSB-chromatin association [14].

**Figure 4:**
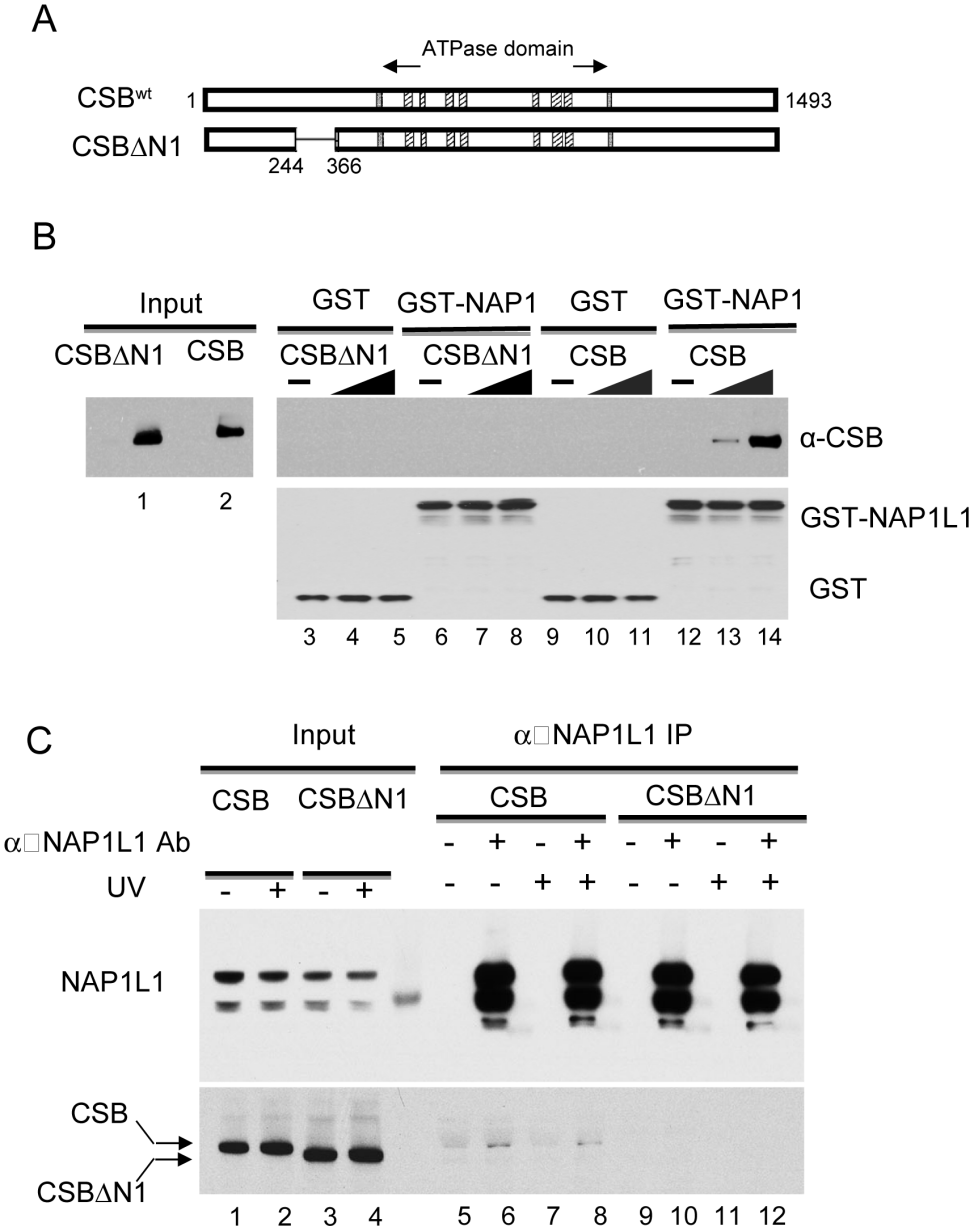
The N1 Region of CSB Is Critical for NAP1L1 Interaction. (A) Schematic illustration of CSB and CSBΔN1 used in assays. (B) Western blot analysis of GST pull-down assays to examine CSB and NAP1L1 interactions. Purified CSB or CSBΔN1 were mixed with immobilized GST or GST-NAP1L1. Western blots showing levels of input CSB and CSBΔN1 as well as their binding to GST-NAP1L1. (C) Immunoprecipitation using an anti-NAP1L1 antibody to examine the CSB- and CSBΔN1-NAP1L1 interactions in cells, with or without UV treatment (100 J/m^2^).

We found that removing amino acids 245–365 (hereafter called the N1 region) disrupted the CSB-NAP1L1 interaction. Bacterially expressed GST or a GST-NAP1L1 fusion protein was immobilized on glutathione beads and mixed with CSB and CSBΔN1 proteins purified from SF9 cells. As shown in Figure 4B, deleting the N1 region of CSB significantly diminished but did not abolish the interaction between CSB and NAP1L1 (compare lanes 6–8 to 12–14).

We also examined the interaction of CSBΔN1 and NAP1L1 *in vivo*. Co-immunoprecipitation experiments were performed using CSB-deficient CS1AN-Sv cells stably expressing CSB or CSBΔN1. As shown in Figure 4C, the amount of CSBΔN1 that co-purified with NAP1L1 was substantially diminished relative to CSB.

**Figure 5:**
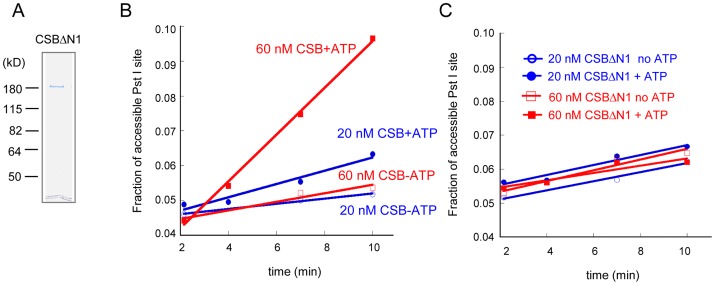
The N1 Region of CSB Is Critical for Chromatin Remodeling. (A) Coomassie stained gel of purified CSBΔN1 used in B–C. (B–C) Restriction enzyme accessibility assays were carried out in the presence of ∼70 nM of 240 bp nucleosomes and limiting amounts of CSB (B) or CSBΔN1 (C). (B) CSB remodeled nucleosomes in an ATP-dependent manner with a rate of 0.12 nM Pst I site cutting/min at 20 nM CSB and 0.42 nM Pst I site cutting/min at 60 nMCSB. (C) Using the same concentrations of CSBΔN1, no ATP-dependent increase in Pst I accessibility was detected.

We next determined the extent to which CSBΔN1 remodeled nucleosomes by performing side-by-side remodeling assays under conditions of limiting amounts of remodeler. When substrate, ATP and Pst I were in excess, both 20 nM and 60 nM CSB demonstrated significant remodeling activity (Figure 5B); however, under the same conditions, CSBΔN1 did not reveal substantial remodeling activity above background (Figure 5C). This diminished remodeling activity is not due to diminished CSBΔN1-nucleosome interaction, as ATPase assays revealed that CSBΔN1 and CSB interacted with the nucleosome substrate equally well (Figure S6). Taken together, these results indicated that residues 245–365 of CSB are critical for both NAP1-like histone chaperone interaction and robust ATP-dependent chromatin remodeling activity.

**Figure 6:**
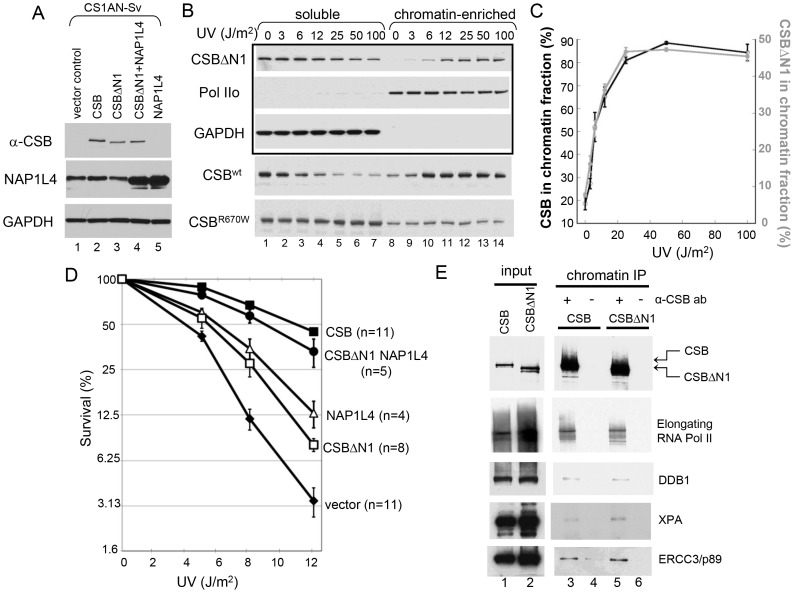
CSB Has Nucleosome Remodeling-Dependent and -Independent Functions in Transcription-Coupled DNA Repair. (A) Western blot showing transgene expression levels of cell lines used in B–E. GAPDH was used as a loading control. Cell lines expressing different transgenes are as indicated at the top of blots, and antibodies used for probing are indicated to the left. (B) UV-induced chromatin association of CSB and CSB derivatives. Cells were irradiated with different UV doses. After 1-hour recovery, lysates were prepared and divided into soluble and chromatin-enriched fractions. Antibodies used in western blot analyses are as indicated to the left. The upper three rows in the boxed area contain samples from CSBΔN1 expressing cells. The lower two rows contain samples from CSB and CSB^R670W^ expressing CS1AN-Sv, used as positive and negative controls, respectively [14]. (C) Quantification of the data in (B), showing the percentage of CSB (black, left Y-axis) and CSBΔN1 (grey, right Y-axis) that co-fractionated with chromatin as a function of UV dose. Shown are averages from three independent experiments +/− SEM. (D) Clonogenic cell-survival assays. CS1AN-Sv cell lines expressing the indicated proteins were treated with 0, 5, 8 or 12 J/m^2^ of UV light. Plotted is the percent survival versus UV dose. Shown are averages +/− SEM. n is number of experiments performed for each cell line. (E) ChIP-western analysis of CSB- and CSBΔN1-interacting proteins. CS1AN-Sv cells expressing CSB or CSBΔN1 were irradiated with 25 J/m^2^ of UV light, allowed to recover for one hour and then subjected to ChIP using anti-CSB antibodies. Immunoprecipitated material was reverse-crosslinked and analyzed by western blot using the indicated antibodies.

To determine if CSBΔN1 could respond appropriately to DNA damage, we examined whether CSBΔN1 became stably associated with chromatin upon UV irradiation [14]. CSBΔN1 was expressed in the CSB-deficient cell line, CS1AN-Sv (Figure 6A), and the response of this CSB derivative to UV-induced DNA lesions was examined. As shown in Figure 6B–6C, like CSB, the majority of CSBΔN1 was in the soluble fraction under conditions of normal cell growth and became chromatin associated upon UV irradiation. To determine if the UV-induced response we observed for CSBΔN1 was similar to that of CSB, we quantified the data shown in Figure 6B [14]. As shown in Figure 6C, CSB and CSBΔN1 demonstrated identical trends with respect to UV-induced chromatin association, saturating at 25 J/m^2^. Together, these results revealed that CSBΔN1 retained the ability to associate with chromatin in a UV-dependent manner.

### The ATP–Dependent Chromatin Remodeling Activity of CSB Is Required for Transcription-Coupled DNA Repair

Since CSBΔN1 becomes chromatin-associated upon UV treatment like CSB, but is defective for chromatin remodeling, we could now use CSBΔN1 as a molecular tool to dissect the importance of ATP-dependent chromatin remodeling by CSB in transcription-coupled DNA repair. Accordingly, we determined if CSBΔN1 could complement the UV-sensitivity phenotype associated with loss-of-CSB activity in CS1AN-Sv cells (Figure 6D). As expected, wild-type CSB substantially rescued the UV sensitivity phenotype. However, rescue by the CSBΔN1 protein was only marginal, indicating that the N1 region is important for CSB function in transcription-coupled DNA repair (Figure 6D).

Given that NAP1L1 and NAP1L4 interact with CSB, we also examined the impact of the NAP1-like histone chaperones on the transcription-coupled DNA repair process. To accomplish this, we sought to examine the effect that these histone chaperones have on the UV-sensitivity phenotype of CS1AN-Sv when overexpressed. The expression of NAP1L1, however, seemed to be tightly regulated, as we could at best increase the amount of NAP1L1 in CS1AN-Sv cells by about 50%. In contrast, we were able to generate a stable CS1AN-Sv cell line that overexpressed NAP1L4 to about 3-fold of the endogenous level (Figure 6A); therefore, we focused our attention on cells overexpressing NAP1L4. Interestingly, we found that NAP1L4 overexpression partially rescued the UV-sensitivity phenotype (Figure 6D). Strikingly, overexpressing NAP1L4 in CSBΔN1 expressing CS1AN-Sv cells rescued the UV-sensitivity to a level close to that observed with wild-type CSB. This result demonstrated allele-specific complementation and indicates that overexpressing NAP1L4 compensates for the diminished affinity of NAP1L4 for CSBΔN1, observed *in vivo* and *in vitro* (Figure 6D).

### CSB–Mediated Recruitment of DNA Repair Proteins and Chromatin Remodeling Are Separable Activities

The association of CSB with lesion-stalled transcription is a prerequisite for recruitment of the DNA repair machinery [28]. In this study, we demonstrated the importance of chromatin remodeling by CSB in transcription-coupled DNA repair. We next wished to determine whether recruitment of the DNA repair machinery by CSB is dependent upon CSB's chromatin remodeling activity. Chromatin remodeling might be necessary to expose DNA for factor binding. Alternatively, repair protein recruitment might be independent of CSB-mediated chromatin remodeling and, instead, rely on other mechanisms, such as protein-protein interaction.

To dissect the function of CSB's chromatin remodeling activity in DNA repair, we performed a side-by-side comparison of UV-induced protein factor recruitment in CS1AN-Sv cells expressing either wild-type CSB or the remodeling-defective CSBΔN1 protein, using ChIP-western analyses [28]. Given that both CSB and CSBΔN1 associate with chromatin in a UV-dependent manner, we found, as expected, that both CSB and CSBΔN1 co-purified with elongating RNA polymerase II, presumably stalled at sites of UV-induced DNA lesions (Figure 6E). Interestingly, we found that the DNA damage-binding protein 1 (DDB1), the Xeroderma pigmentosum group A-complementing protein (XPA) and the DNA excision repair protein ERCC3 also co-purified with both CSB and CSBΔN1 (Figure 6E and Figure S7). Moreover, these protein associations were UV-dependent and occurred at both high (25 J/m^2^) and low (6 J/m^2^) UV doses (Figure 6E and Figure S7, respectively). These data revealed that CSB-mediated recruitment of components of the transcription-coupled DNA repair machinery to DNA lesion-stalled transcription does not rely upon chromatin remodeling by CSB.

## Discussion

**Figure 7:**
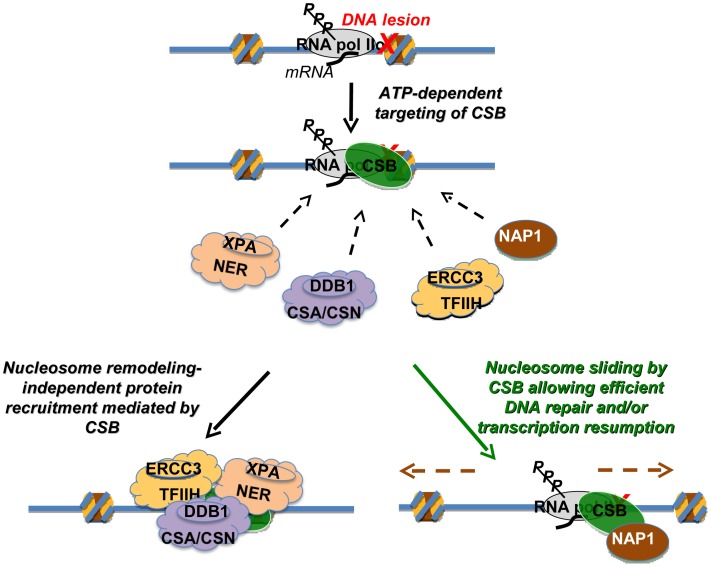
Model Depicting the Distinct Activities of CSB in Transcription-Coupled DNA Repair. The targeting of CSB to lesion-stalled transcription (Top) is a critical and early step in the process of transcription-coupled DNA repair [28]. Once recruited, CSB has two separable activities. Lower left: one activity is protein factor recruitment that occurs independently of nucleosome repositioning. Recruitment includes components of the nucleotide excision repair complex, the CSA/CSN E3 ligase complex and the TFIIH transcription elongation complex [28]. Direct interactions between CSB and the NAP1-like chaperones suggest that CSB also recruits NAP1L1 and NAP1L4 to sites of lesion-stalled transcription. Lower right: a second activity is dependent upon nucleosome repositioning. In conjunction with the NAP1-like histone chaperones, CSB slides nucleosomes to allow efficient DNA repair and/or resumption of transcription after repair.

In this study, we found that CSB is a relatively inefficient remodeler that slides nucleosomes away from DNA ends and creates an array of remodeled products containing histone octamers at different positions (Figure 1). This weak remodeling activity is similar to that of the Drosophila ISWI remodeler, which interacts with the dAcf1 protein to form the dACF complex; in the absence of Acf1, the ISWI remodeler displays only 3% of the activity that it does when in complex with Acf1 [29]. In our study, we identified the human histone chaperones NAP1L1 and NAP1L4 as new CSB-binding partners and found that each of these proteins greatly accelerated chromatin remodeling by CSB (Figure 2). Strikingly, in the presence of either of these histone chaperones, one predominant remodeled product containing a centrally localized nucleosome was created (Figure 3 and Figure S5). By mapping functional domains and analyzing CSB derivatives, we found that CSB-mediated recruitment of some critical components of the DNA repair machinery (i.e. XPA, DDB1 and ERCC3) occurs independently of chromatin remodeling by CSB (Figure 6 and Figure S7). Our finding that co-expression of the remodeling-defective CSBΔN1 protein and NAP1L4 can rescue the UV sensitivity of CS1AN-Sv cells to a level similar to wild-type CSB, supports the notion that the protein recruitment and remodeling activities of CSB are separable (Figure 7). In this case, NAP1L4 overexpression would compensate for the diminished remodeling activity of CSBΔN1, while CSBΔN1 would maintain the capacity to recruit essential repair factors.

How then might the ATP-dependent chromatin remodeling activity of CSB be utilized in the DNA repair mechanism? Given that the remodeling defective CSBΔN1 protein alone can only partially rescue loss-of-CSB function, a key role for chromatin remodeling by CSB may be to make sure that the overall repair process occurs as efficiently as possible, by decreasing nucleosome barriers. During normal transcription as RNA polymerase translocates, histone octamers can be completely displaced from the DNA template or partially disassembled through loss of an H2A/H2B dimer [30]. If, however, a bulky DNA lesion is located within the bounds of a histone octamer, that lesion could conceivably stall an RNA polymerase before the polymerase has a chance to displace the nucleosome from its path. Nucleosomes that surround a stalled RNA polymerase could create barriers to any step of the repair mechanism, from RNA polymerase displacement, through nucleotide incision and excision, to DNA re-synthesis and transcription resumption [6], [31], [32]. Although *in vitro* assays indicate that neither RNA polymerase displacement nor CSB activity is required for the 3′ and 5′ incisions that are needed for the excision of lesion-containing DNA [33], these *in vitro* studies utilized naked DNA templates that do not reflect all the complexities of transcription-coupled repair that occur within the context of chromatin *in vivo*. Accordingly, the combined activities of CSB and NAP1-like chaperones might mobilize histones, nucleosomes and/or stalled RNA polymerase to allow efficient coupling of transcription and DNA repair. In agreement with this notion, the SWI/SNF chromatin-remodeling complex has been shown to increase the overall efficiency of global genome excision repair, revealing the importance of chromatin dynamics in nucleotide excision repair in general [34], [35]. In addition to nucleosome repositioning, CSB has been shown to possess other ATP-dependent and ATP-independent activities, such as DNA wrapping and strand annealing [11], [12]. Consequently, these other activities are also likely to play important roles in transcription-coupled DNA repair.

The mutation frequency decline protein, Mfd, is the factor that couples transcription with repair in bacteria, and the critical functions of this protein are to displace stalled RNA polymerase and to initiate repair protein recruitment [36]. CSB also initiates repair protein recruitment, but CSB alone has not been found capable of displacing a stalled RNA polymerase [9]. However, a major difference between transcription-coupled DNA repair in eukaryotes and prokaryotes is that, in addition to a stalled RNA polymerase, nucleosomes can also create barriers to the repair process. Our results indicate that nucleosomes repositioning by CSB is critical to the coupling of DNA repair with transcription in eukaryotes.

The enhancement of CSB-mediated chromatin remodeling by NAP1-like histone chaperones adds a new dimension to our understanding of the ways in which ATP-dependent chromatin remodelers and histone chaperones can collaborate. Histone chaperones have been found to function with ATP-dependent chromatin remodelers in the exchange of histones and histone variants as well as in chromatin assembly (ACF, CHD1) and disassembly (RSC) [8], [16], [17], [37]. Results from this study now reveal that histone chaperones can cooperate with ATP-dependent chromatin remodelers to increase the efficiency of nucleosome repositioning by inefficient remodelers. It remains to be determined whether CSB and the NAP1-like chaperones cooperate in nucleosome assembly/disassembly during normal transcription and transcription-coupled DNA repair.

Several NAP1-like proteins have been identified based on sequence homology [19], and the functions of many are still unknown (Figure S1). Among them, NAP1L2, NAP1L3, NAP1L5 are predominantly expressed in the nervous system, and TSPY is testis-specific [19]. As the highly structured, central NAP domain is sufficient for CSB binding (data not shown), CSB may likely interact with other NAP1-like protein. It is tempting to speculate that aberrant interactions between mutant CSB proteins and tissue specific NAP1-like proteins might contribute to the diverse clinical features associated with Cockayne syndrome.

## Materials and Methods

### Construct Generation, Protein Expression, and GST Pull-Down Assays

Constructs encoding CSB, CSB derivatives, NAP1L1 and NAP1L4 were generated by PCR and cloned into pDONR, pGEX-4T, pFastBac1 or pLenti-PGK vectors using Gateway technology (Invitrogen). GST pull-down assays and protein expression in and purification from bacteria, insect cells and mammalian cells were performed as previously described [10]. hSWI/SNF was purified from HeLa cells stably expressing Flag-Ini1 [38].

### Nucleosome Assembly

DNA fragments of ∼240 bp containing the 601 nucleosome phasing sequence located at different positions, and 202 bp DNA fragments containing the TPT phasing sequence, were assembled into mononucleosome with purified HeLa histones or recombinant human histones, using step-gradient salt dialysis [24], [26]. DNA fragments used for assembly were generated by PCR and body-labeled with [^32^P] α-dATP.

### ATPase and Nucleosome Remodeling Assays

ATP hydrolysis reactions and remodeling assays were carried out in 12 mM Hepes (pH7.9), 10 mM Tris⋅HCl (pH 7.5), 60 mM KCl, 8% glycerol, 4 mM MgCl_2_, 2 mM ATP^.^Mg and 0.02% NP40 at 30°C as described previously [26]. Nucleosomes were used at 2.5 nM, and Pst I was used at 2 U/µl in restriction enzyme accessibility assays shown in all figures except Figure 5. The remodeling rates in all figures except Figure 5 were determined by using an exponential fit of the data points, as described previously [26]. Remodeling reactions shown in Figure 5 were carried out with excess nucleosomes (∼70 nM) over enzyme; the remodeling rates were obtained from initial rates determined by linear fits of the data for the first 10% of cut nucleosomes.

### Cell Culture

CS1AN-Sv cells were maintained in DMEM-F12 with 10% FBS. HeLa cells were cultured in S-MEM with 5% FBS. Stable cell lines were generated by infecting CS1AN-Sv cells with lentivirus (pLenti-PGK, Addgene) expressing different transgenes and selected with 600 µg/ml G418 or 250 µg/ml hygromycin.

### UV Sensitivity, Protein Fractionation, and Protein Interaction Assays

For UV sensitivity assays, cells were irradiated with UV light using a Stratalinker (Agilent). After 10-days of growth, colonies were fixed and stained with crystal violet. For protein fractionation assays, cells were treated with UV light, and lysates were prepared 1 hour after irradiation [14]. For protein interaction assays, chromatin-enriched fractions were subjected to immunoprecipitation with anti-CSB, anti-NAP1L1 (ab33076, Abcam), or anti-NAP1L4 (ab21631, Abcam) antibodies, cross-linked to Protein A/G agarose beads.

### ChIP–Western Analysis

Cells were irradiated with 25 J/m^2^ UV light and allowed to recover for 1 hour, after which cells were fixed with 1% formaldehyde for 10 min. Chromatin IP (ChIP) was performed using anti-CSB antibodies as previously described [14], [28]. Antibodies used for western blot analysis of ChIPed samples were Phospho RNA Pol II (S2) (A300-654A, Bethyl), DDB1 (A300-462A, Bethyl), p89 (SC-293, Santa Cruz) and XPA (X1504, Sigma).

## Supporting Information

Figure S1 Related to Figure 2. Comparison of Human NAP1-like Proteins. (A) Phylogenetic tree showing the evolutionary relationships of the five human NAP1-like proteins. (B) Sequence alignment of human NAP1L1 and NAP1L4 proteins showing fully conserved residues (*), residues with strong similarity (:), and residues with weak similarity (.). Sequence comparisons shown in (A) and (B) were generated with ClustalW2.
(TIF)

Figure S2 Related to Figure 1. Remodeling Activity of CSB on Five Different Mononucleosomal Substrates. (A) Core, (B) C+45, (C) C+120, (D) 45+C+120 and (E) 108+C+120, each with a Pst I site located at position 25, as depicted. Open circles are reactions without ATP. Closed circles are reactions containing ATP; rates for CSB were 0.004, 0.009, 0.011, 0.02 and 0.02 min^−1^ on substrates used in (A–E) respectively.
(TIF)

Figure S3 Related to Figure 1. CSBΔN is a Functional DNA- and Nucleosome-Stimulated ATPase. (A) 15 nM of CSB and (B) 15 nM of CSBΔN were used in ATPase assays with varying amounts of DNA or nucleosomes.
(TIF)

Figure S4 Related to Figure 2. NAP1L1 and NAP1L4 Interact with CSB. (A–B) Summary of NAP1L1 and NAP1L4 data obtained from mass spectrometry. Green, underscored letters indicate identified peptides. Peptide positions and protein coverage are as noted. (C) Nuclei from HeLa cells with or without UV treatment (100 J/m^2^) were isolated and subjected to immunoprecipitation using an anti-NAP1L1 antibody. Immunoprecipitates were resolved by SDS-PAGE and probed with antibodies shown to the right.
(TIF)

Figure S5 Related to Figure 3. Mapping CSB and CSB/NAP1L1 Remodeled Products. (A) Electrophoretic mobility-shift assays to analyze ACF-remodeled nucleosome structure. NAP1L1 does not alter ACF-mediated remodeling of an end-positioned mononucleosome with a 91 bp DNA overhang. (B) Restriction enzyme mapping of purified, MNase-resistant DNA fragments. Lanes 1–4 indicate that the nucleosomal substrate used had the histone octamer covering the left 150 bps of the C+91 DNA. Lanes 5–8 reveal that, after CSB remodeling, ∼60% of the nucleosomes still had histone octamers covering the left 150 bp of DNA. There are three additional nucleosome species; each representing about 10% of the total population, and each off set by about 15 bp to the left. Lanes 9–12 reveal that ∼80% of CSB/NAP1L1 remodeled products cover the central 150 bp of the DNA fragment. (C) Representation of remodeled products generated by CSB or by CSB and NAP1-like chaperones. On its own, CSB does not remodel nucleosomes efficiently. Less than 40% of the substrate is remodeled at best, and the nucleosome positions of the remodeled products are heterogeneous. Right: NAP1-like histone chaperones enable CSB to remodel nucleosomes robustly; together, these proteins remodel more than 80% of the nucleosomal substrate and create centrally localized nucleosomes. The NAP1-like histone chaperones, on their own, do not reposition nucleosomes.
(TIF)

Figure S6 Related to Figure 5. Apparent K_M_ Determination of CSB and CSBΔN1 for Nucleosomes. (A) CSB was used in ATP hydrolysis assays in the presence of varying amounts of 240 bp mononucleosomes. Nucleosome concentrations were 240, 80, 27, 9, 3 nM, and buffer only (from top to bottom). (B) Same as in (A), except that CSBΔN1 was used in ATPase assays. (C) Rate constants determined from ATPase assays were plotted against nucleosome concentrations to determine the K_M_ of CSB and CSBΔN1 for nucleosomes. These results revealed that CSB and CSBΔN1 interact with nucleosomes equally well.
(TIF)

Figure S7 Related to Figure 6. CSBΔN1, a Mutant Defective in Nucleosome Repositioning, can Recruit Components of the Transcription-Coupled DNA Repair Machinery. CS1AN-sv cells stably expressing CSB or CSBΔN1 were mock treated or treated with UV irradiation (6 J/m^2^). After a one-hour recovery, cells were extracted with 0.5% triton X-100 to remove soluble proteins, and the chromatin-enriched fraction was crossed linked and subjected to ChIP. CSB-interacting protein were immunoprecipitated with an anti-CSB antibody. Antibodies used for western blot analysis are shown to the right.
(TIF)
