# Supplementary figures and images for: ATP-Dependent Chromatin Remodeling by Cockayne Syndrome Protein B and NAP1-Like Histone Chaperones Is Required for Efficient Transcription-Coupled DNA Repair
Source: PLoS Genet. 2013 Apr 18;9(4):e1003407. doi: 10.1371/journal.pgen.1003407 (PMC3630089; doi:10.1371/journal.pgen.1003407)

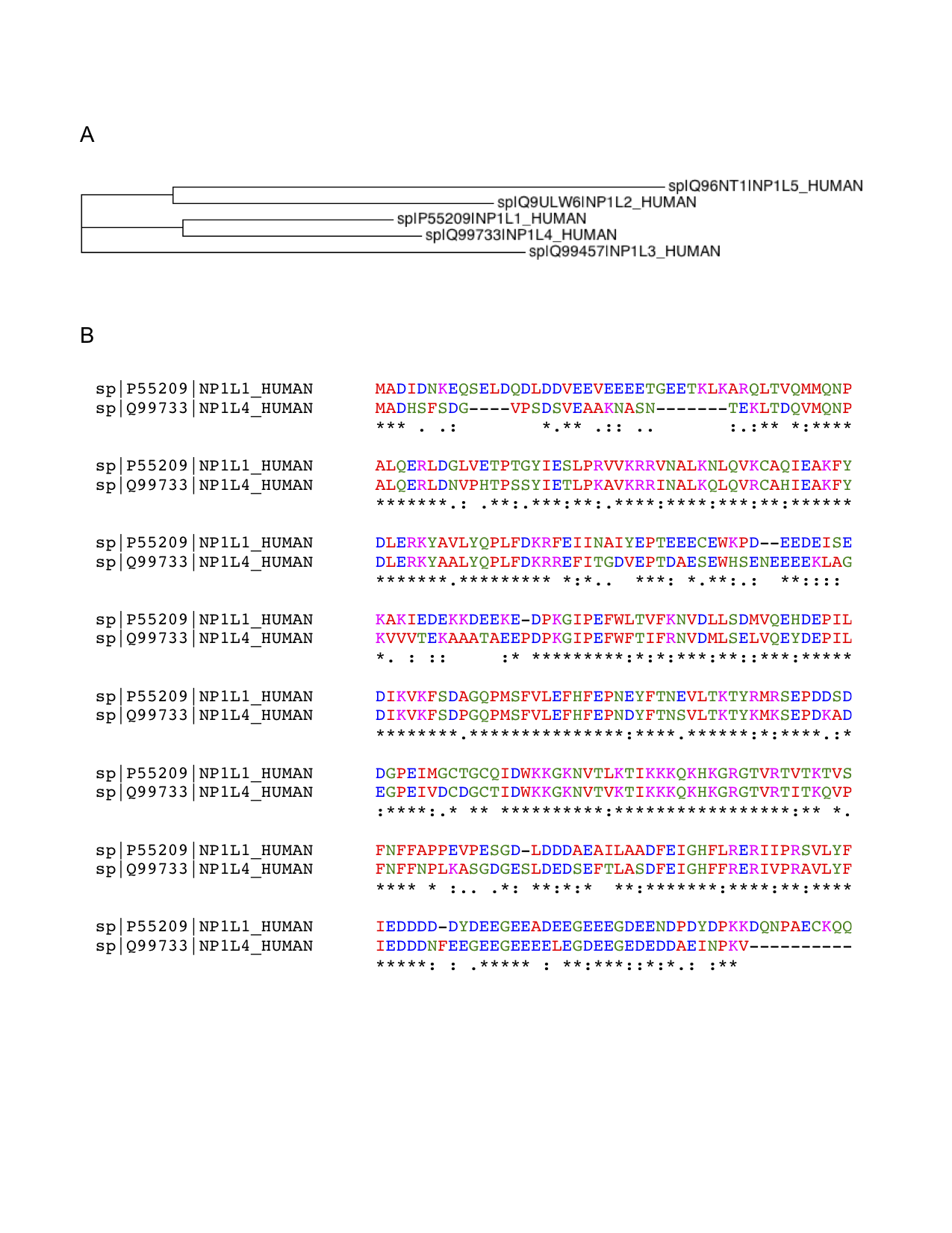

Supplement: Figure S1 — Related to Figure 2. Comparison of Human NAP1-like Proteins. (A) Phylogenetic tree showing the evolutionary relationships of the five human NAP1-like proteins. (B) Sequence alignment of human NAP1L1 and NAP1L4 proteins showing fully conserved residues (*), residues with strong similarity (:), and residues with weak similarity (.). Sequence comparisons shown in (A) and (B) were generated with ClustalW2. (TIF) [file pgen.1003407.s001.tif]

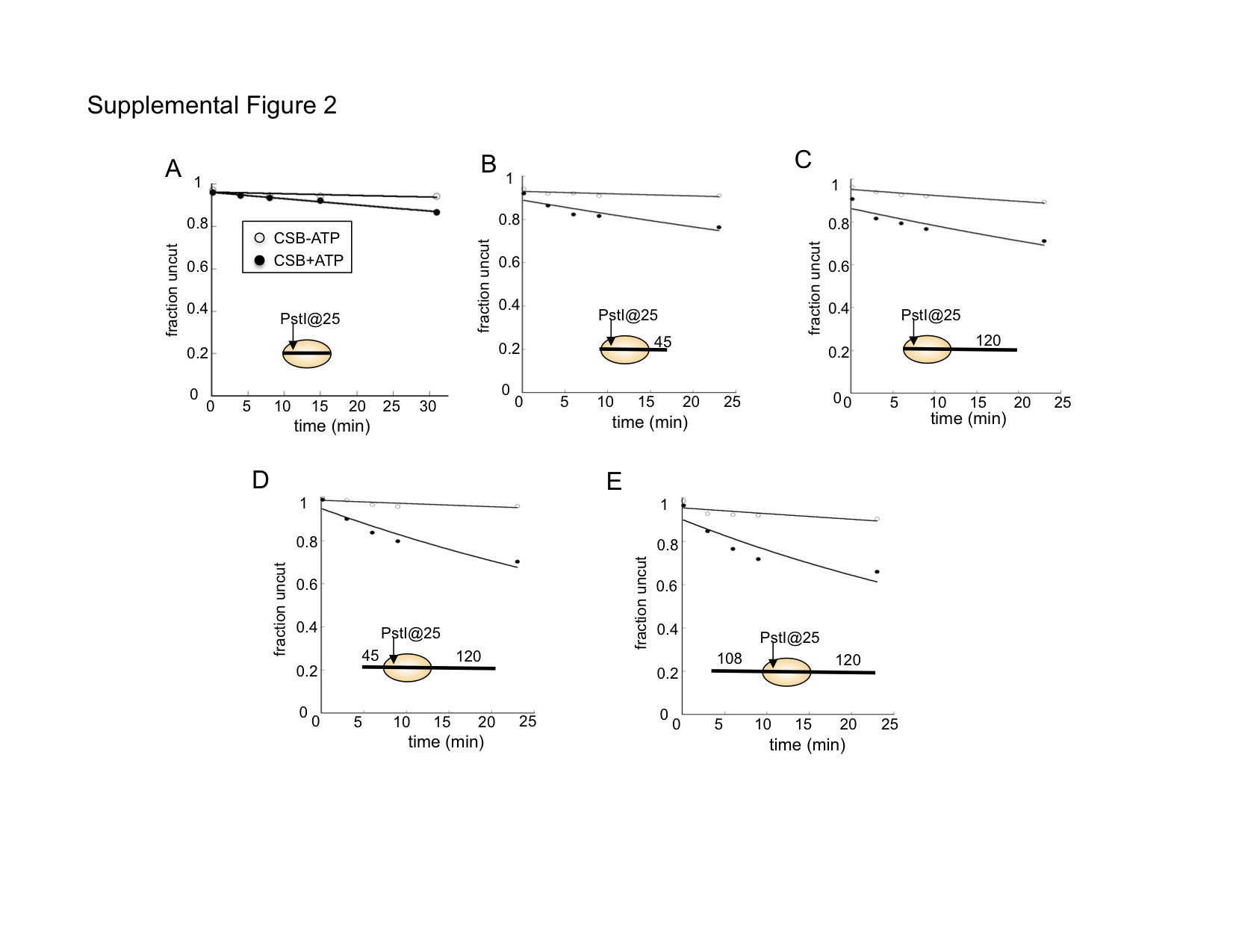

Supplement: Figure S2 — Related to Figure 1. Remodeling Activity of CSB on Five Different Mononucleosomal Substrates. (A) Core, (B) C+45, (C) C+120, (D) 45+C+120 and (E) 108+C+120, each with a Pst I site located at position 25, as depicted. Open circles are reactions without ATP. Closed circles are reactions containing ATP; rates for CSB were 0.004, 0.009, 0.011, 0.02 and 0.02 min−1 on substrates used in (A–E) respectively. (TIF) [file pgen.1003407.s002.tif]

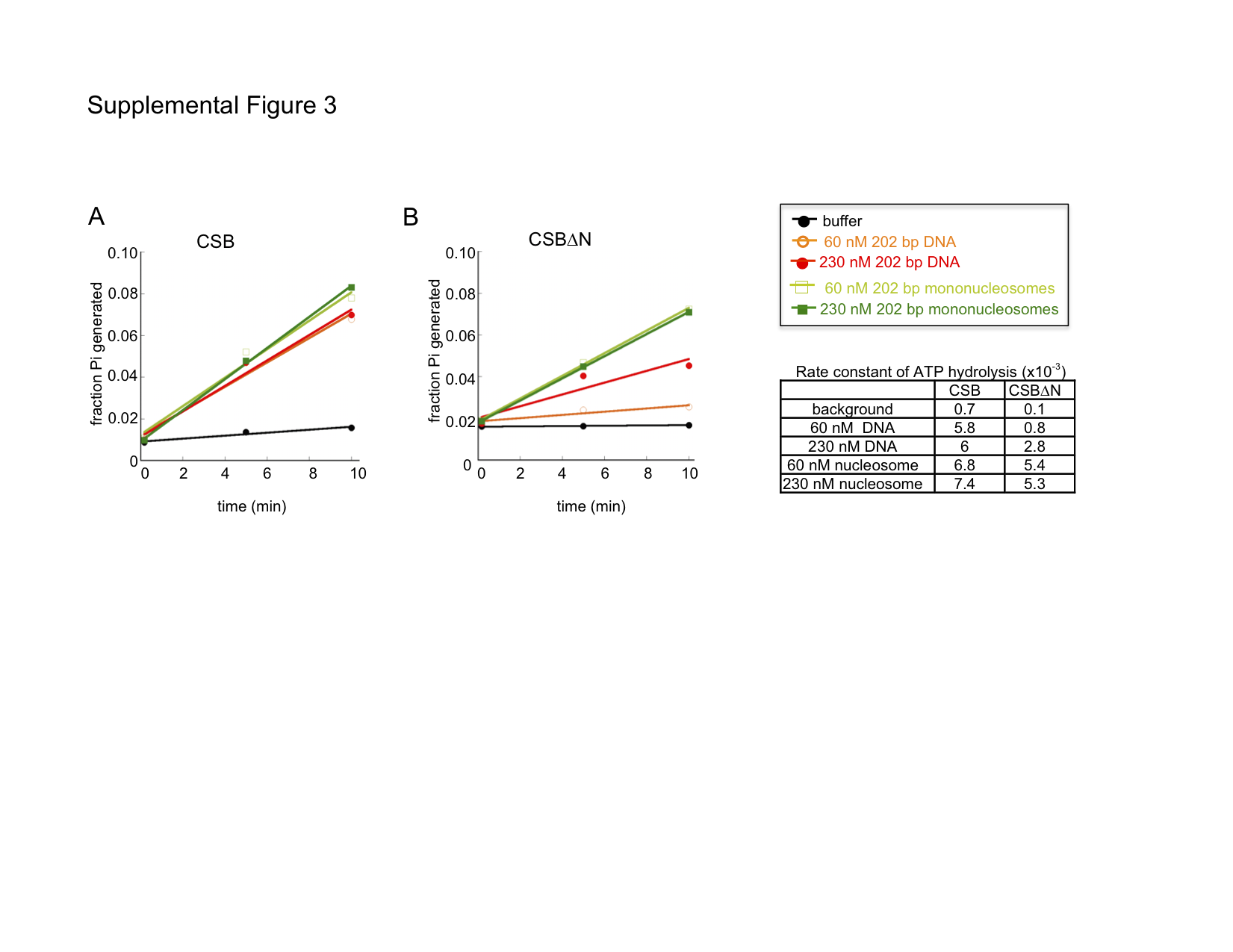

Supplement: Figure S3 — Related to Figure 1. CSBΔN is a Functional DNA- and Nucleosome-Stimulated ATPase. (A) 15 nM of CSB and (B) 15 nM of CSBΔN were used in ATPase assays with varying amounts of DNA or nucleosomes. (TIF) [file pgen.1003407.s003.tif]

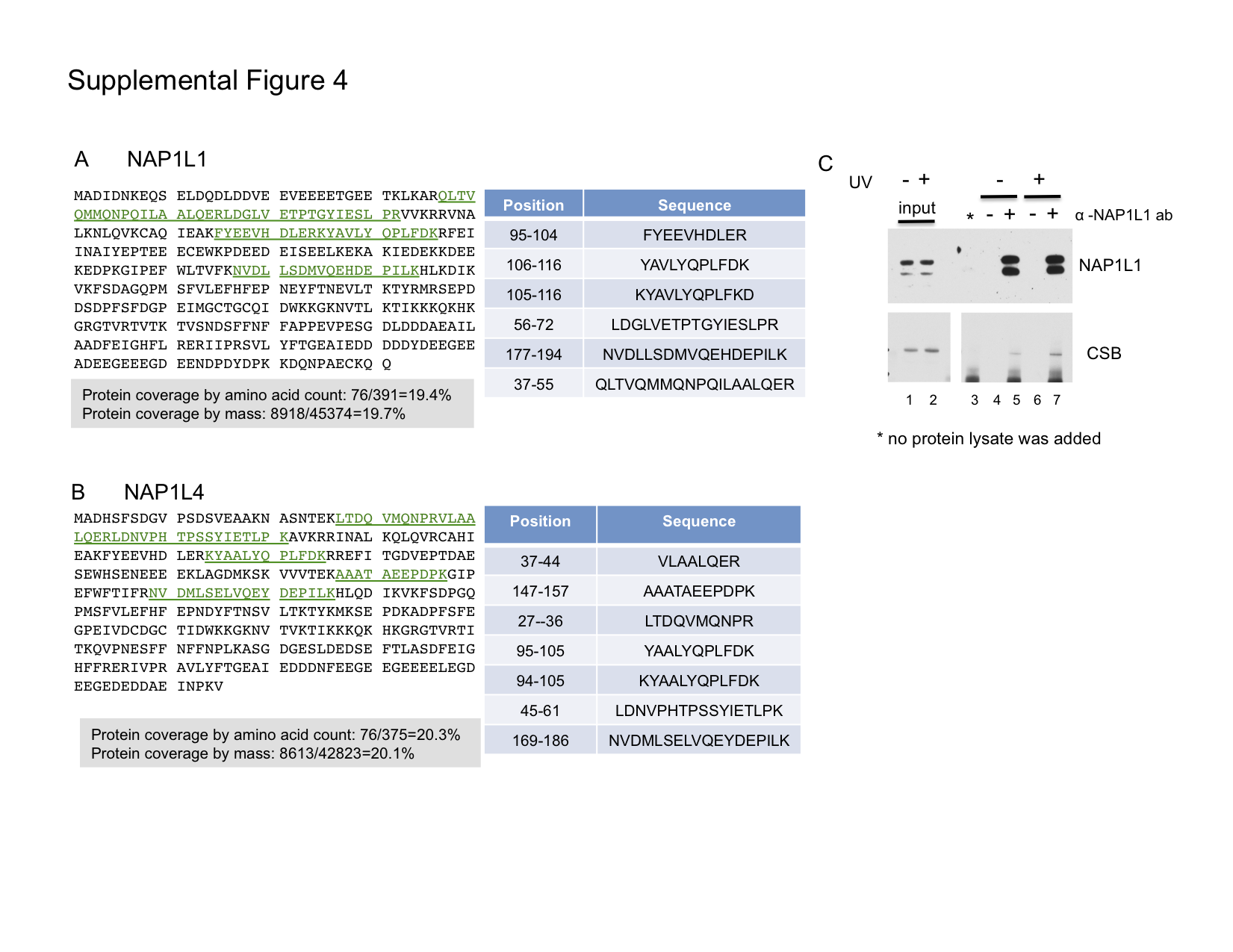

Supplement: Figure S4 — Related to Figure 2. NAP1L1 and NAP1L4 Interact with CSB. (A–B) Summary of NAP1L1 and NAP1L4 data obtained from mass spectrometry. Green, underscored letters indicate identified peptides. Peptide positions and protein coverage are as noted. (C) Nuclei from HeLa cells with or without UV treatment (100 J/m2) were isolated and subjected to immunoprecipitation using an anti-NAP1L1 antibody. Immunoprecipitates were resolved by SDS-PAGE and probed with antibodies shown to the right. (TIF) [file pgen.1003407.s004.tif]

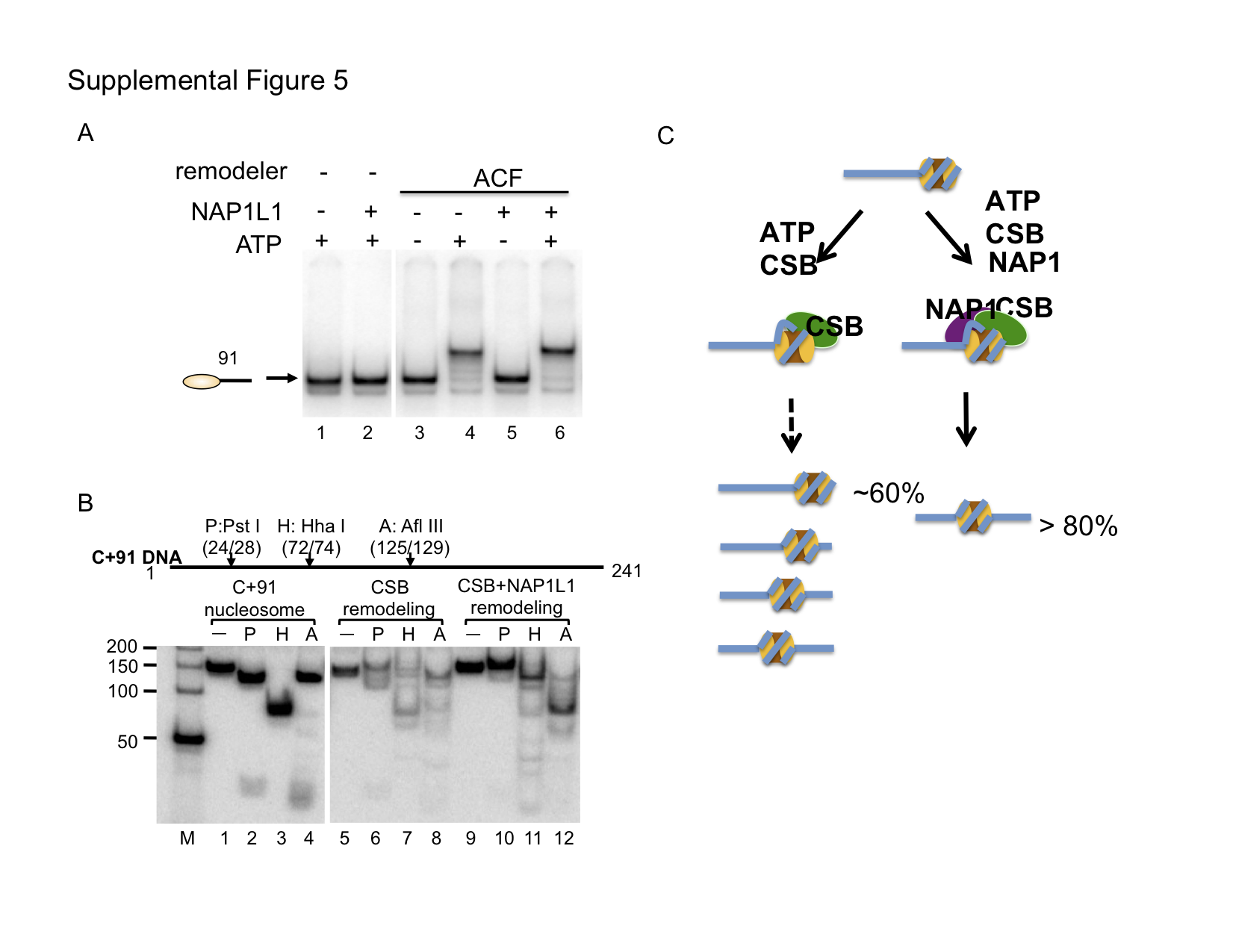

Supplement: Figure S5 — Related to Figure 3. Mapping CSB and CSB/NAP1L1 Remodeled Products. (A) Electrophoretic mobility-shift assays to analyze ACF-remodeled nucleosome structure. NAP1L1 does not alter ACF-mediated remodeling of an end-positioned mononucleosome with a 91 bp DNA overhang. (B) Restriction enzyme mapping of purified, MNase-resistant DNA fragments. Lanes 1–4 indicate that the nucleosomal substrate used had the histone octamer covering the left 150 bps of the C+91 DNA. Lanes 5–8 reveal that, after CSB remodeling, ∼60% of the nucleosomes still had histone octamers covering the left 150 bp of DNA. There are three additional nucleosome species; each representing about 10% of the total population, and each off set by about 15 bp to the left. Lanes 9–12 reveal that ∼80% of CSB/NAP1L1 remodeled products cover the central 150 bp of the DNA fragment. (C) Representation of remodeled products generated by CSB or by CSB and NAP1-like chaperones. On its own, CSB does not remodel nucleosomes efficiently. Less than 40% of the substrate is remodeled at best, and the nucleosome positions of the remodeled products are heterogeneous. Right: NAP1-like histone chaperones enable CSB to remodel nucleosomes robustly; together, these proteins remodel more than 80% of the nucleosomal substrate and create centrally localized nucleosomes. The NAP1-like histone chaperones, on their own, do not reposition nucleosomes. (TIF) [file pgen.1003407.s005.tif]

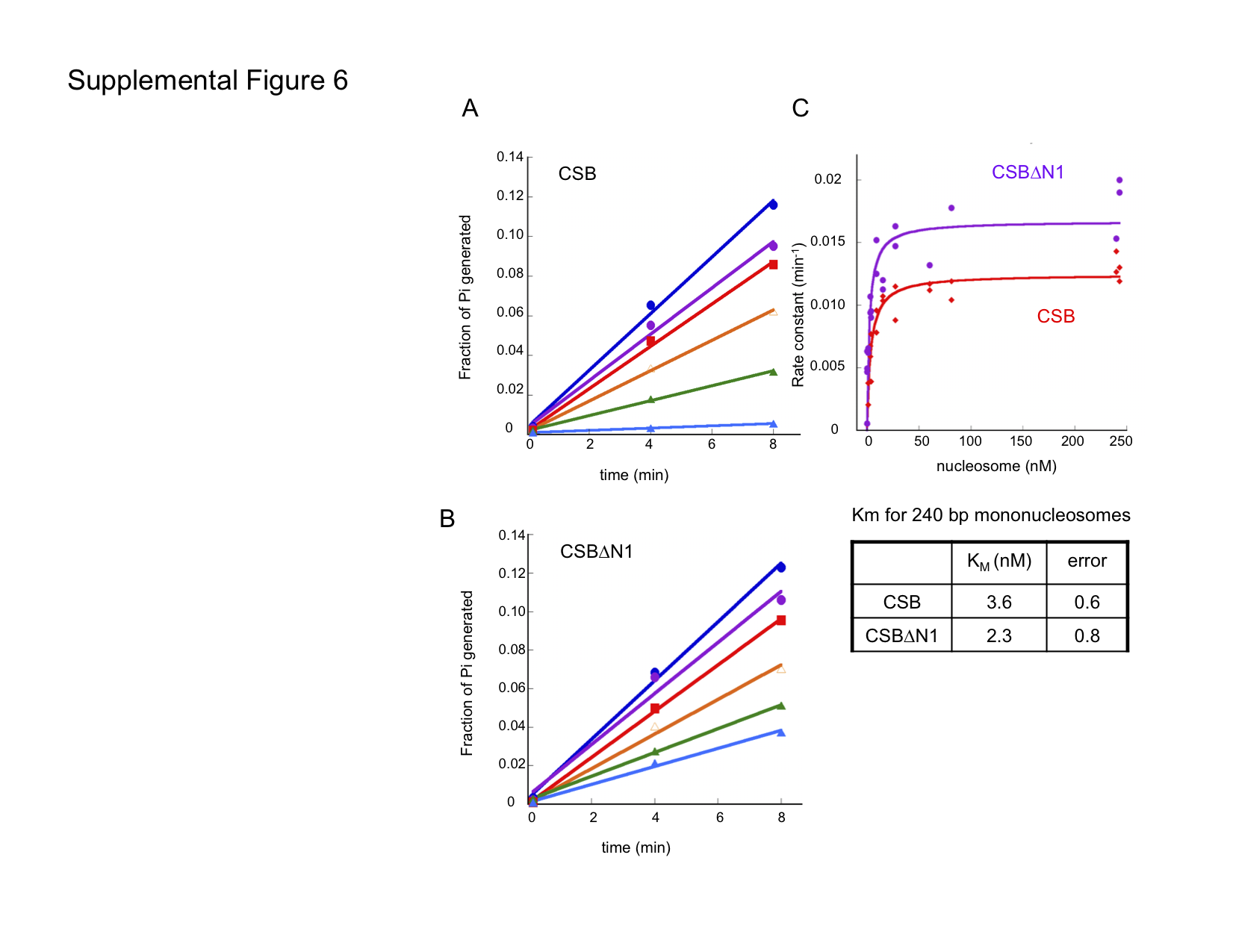

Supplement: Figure S6 — Related to Figure 5. Apparent KM Determination of CSB and CSBΔN1 for Nucleosomes. (A) CSB was used in ATP hydrolysis assays in the presence of varying amounts of 240 bp mononucleosomes. Nucleosome concentrations were 240, 80, 27, 9, 3 nM, and buffer only (from top to bottom). (B) Same as in (A), except that CSBΔN1 was used in ATPase assays. (C) Rate constants determined from ATPase assays were plotted against nucleosome concentrations to determine the KM of CSB and CSBΔN1 for nucleosomes. These results revealed that CSB and CSBΔN1 interact with nucleosomes equally well. (TIF) [file pgen.1003407.s006.tif]

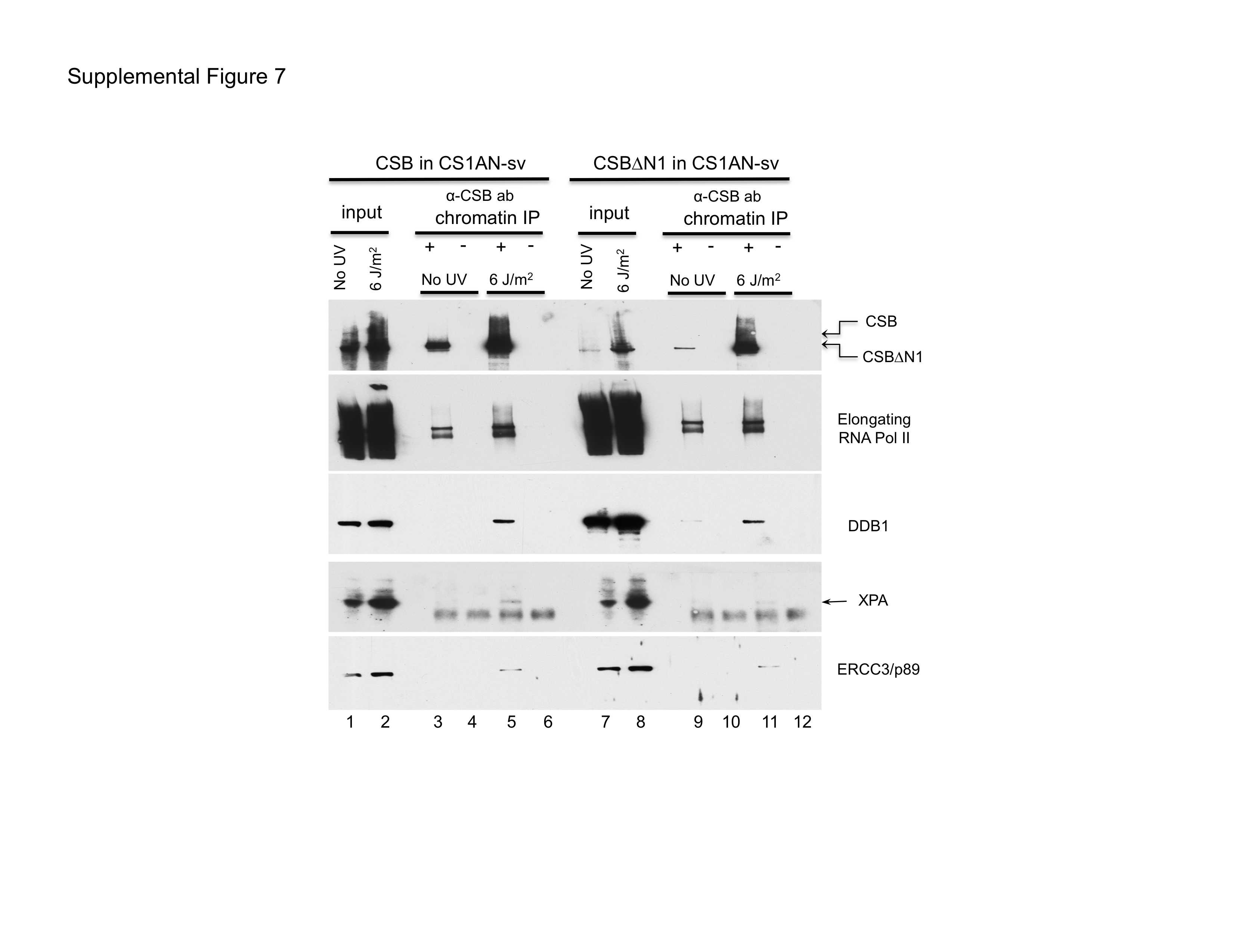

Supplement: Figure S7 — Related to Figure 6. CSBΔN1, a Mutant Defective in Nucleosome Repositioning, can Recruit Components of the Transcription-Coupled DNA Repair Machinery. CS1AN-sv cells stably expressing CSB or CSBΔN1 were mock treated or treated with UV irradiation (6 J/m2). After a one-hour recovery, cells were extracted with 0.5% triton X-100 to remove soluble proteins, and the chromatin-enriched fraction was crossed linked and subjected to ChIP. CSB-interacting protein were immunoprecipitated with an anti-CSB antibody. Antibodies used for western blot analysis are shown to the right. (TIF) [file pgen.1003407.s007.tif]
